# Plant bZIP Transcription Factors Responsive to Pathogens: A Review

**DOI:** 10.3390/ijms14047815

**Published:** 2013-04-10

**Authors:** Murilo S. Alves, Silvana P. Dadalto, Amanda B. Gonçalves, Gilza B. De Souza, Vanessa A. Barros, Luciano G. Fietto

**Affiliations:** Department of Biochemistry and Molecular Biology, Federal University of Viçosa, Viçosa, Minas Gerais 36570000, Brazil; E-Mails: murilobqi@yahoo.com.br (M.S.A.); sil_dadalto@yahoo.com.br (S.P.D.); amandabonoto@gmail.com (A.B.G.); gilzab18@gmail.com (G.B.D.S.); vanessa.barros.ufv@gmail.com (V.A.B.)

**Keywords:** plant defense, biotic stress, bZIP transcription factors

## Abstract

Transcription factors of the basic leucine zipper (bZIP) family control important processes in all eukaryotes. In plants, bZIPs are master regulators of many central developmental and physiological processes, including morphogenesis, seed formation, abiotic and biotic stress responses. Modulation of the expression patterns of bZIP genes and changes in their activity often contribute to the activation of various signaling pathways and regulatory networks of different physiological processes. However, most advances in the study of plant bZIP transcription factors are related to their involvement in abiotic stress and development. In contrast, there are few examples of functional research with regard to biotic stress, particularly in the defense against pathogens. In this review, we summarize the recent progress revealing the role of bZIP transcription factors in the biotic stress responses of several plant species, from *Arabidopsis* to cotton. Moreover, we summarize the interacting partners of bZIP proteins in molecular responses during pathogen attack and the key components of the signal transduction pathways with which they physically interact during plant defense responses. Lastly, we focus on the recent advances regarding research on the functional role of bZIPs in major agricultural cultivars and examine the studies performed in this field.

## 1. Introduction

Plants are sessile organisms and, as such, are constantly exposed to stress conditions, including cold, salinity, drought, flood, high temperature, toxic heavy metals, pathogens and herbivores [1]. These factors restrict the growth and productivity of many crops. The induced expression of various stress responsive genes occurs under these conditions, contributing to minimizing the effects caused by stress. These genes can be classified into two groups: the first group includes genes related to cell metabolism and stress tolerance, and the second group consists of regulatory genes that encode protein kinases or phosphatases and transcription factors [2].

Transcription factors are DNA-binding proteins that interact with other components of the transcriptional machinery to recruit or block the access of RNA polymerase to the gene promoter [3]. In some cases, a transcription factor can regulate the expression of multiple genes, and this regulation is possible, due to the recognition of similar sequences in different promoters [4].

Several families of transcription factors have been reported to be involved in plant defense against pathogens. Among the most studied during the last decade are the WRKY transcription factors and MYB transcription factors; in addition, a few studies have reported the role of transcription factors containing a basic leucine zipper domain (bZIP) [5]. The WRKY proteins belong to a transcription factor family that is exclusive to plants, presenting in its structure one or two domains containing the sequence, WRKYGQK [6], a domain that typically binds a *cis*-element called a W box. WRKY proteins are responsive to a wide range of pathogens and defense hormones, such as salicylic acid (SA) [6], whereas MYB proteins appear to be linked to the response to various types of stress, such as ultraviolet rays, injury, anaerobic conditions and pathogens [5].

The family of transcription factors containing a bZIP domain is among the largest families of transcription factors in plants, though these proteins are found in all eukaryotes. In plants, these factors regulate genes in response to abiotic stress, seed maturation, flower development and pathogen defense [7].

**Figure 1:**
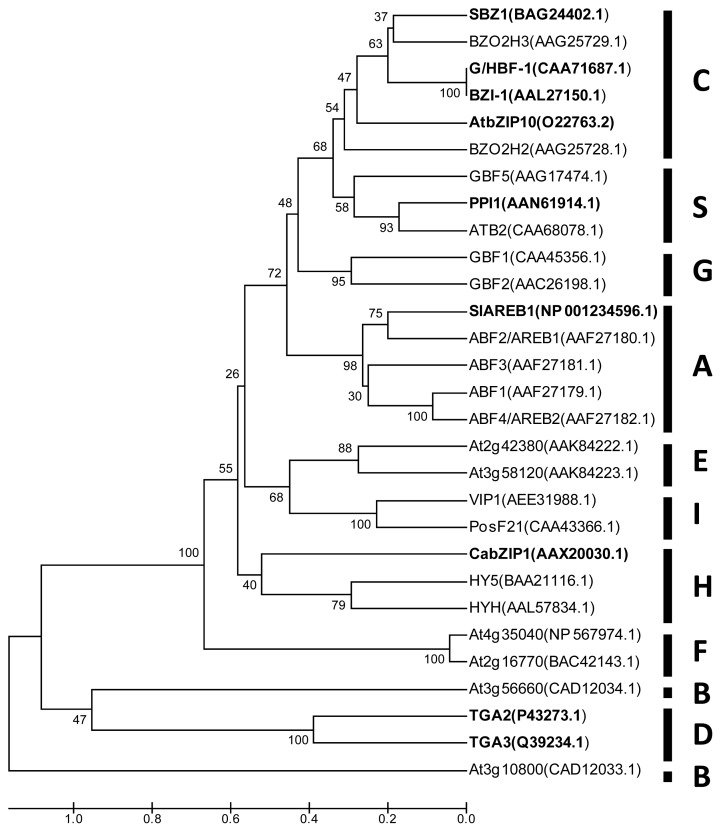
Relatedness of bZIP proteins from different plant species. The multiple alignment was generated using MUSCLE and the phylogenetic tree was built with the MEGA5 software using the UPGMA method (the numbers at the nodes indicate the bootstrap scores). The bZIP proteins were separated into ten (10) groups, as indicated [7]. The protein accession numbers are indicated in parentheses. The proteins responsive to pathogens are highlighted in bold.

Jakoby *et al*. [7] placed the *Arabidopsis* bZIP proteins (AtbZIP) into ten groups: A, B, C, D, E, F, G, H, I and S. Each group has sequence similarity in the basic region and extended features in common, such as the size of the leucine zipper domain and its position in the protein sequence. In soybean, the classification of the bZIP transcription factors (GmbZIPs) was performed by analyzing 47 bZIP sequences together with 75 AtbZIP proteins [4]. As a result, 10 groups were classified, similar to *Arabidopsis*[4]. Because this classification is based on conserved domains, it is expected to be useful for the general functional classification of bZIP proteins of plants. However, as observed in Figure 1, there is no a direct correlation between the arrangement of bZIP domains and protein function. A phylogenetic tree was constructed using the complete amino acid sequences of pathogen-responsive bZIP proteins and proteins related to other environmental responses (Figure 1). The proteins were grouped according to the classification of Jakoby and collaborators [7], whereby some bZIP proteins of known function in the defense against pathogens grouped with proteins that have a function in abiotic stress response, such as the SlAREB1 protein. This result most likely occurred because the function of these proteins is determined by the location or number of bZIP domains in their structure and also by other, not-yet-characterized functional domains.

However, among the various functions of bZIP proteins, defense against pathogens in plants is one of the least studied [5,7]. Accordingly, this review discusses the state-of-the-art research on the role of bZIP transcription factors in response to plant pathogens, addressing the lack of studies on these transfactors in crops of agronomic importance (Table 1).

## 2. Response to Pathogens in Plants: Signaling and bZIP Transcription Factors

During pathogen recognition, plants trigger a defense response via the direct or indirect interaction between the product of the resistance gene (R) and effector proteins produced by the pathogen, a process called immunity triggered by immune effectors (TSI) [22,23]. If there is a lack of corresponding genes, the plant is susceptible to infection and cannot activate defense responses with speed and intensity in relation to the initial event. In addition, plants have a recognition system based on receptors called immunity triggered by pathogen-associated molecular patterns (PTI) [22,23], which confers a low-level resistance to virulent pathogens. Salicylic acid (SA) is an important signal for the activation of PTI [24] and TSI [25–28], and jasmonic acid (JA) and ethylene (ET) also represent important signaling molecules in plant defense. An important aspect of jasmonic acid action is a synergistic interaction with ethylene in the induction of a large group of genes related to defense.

The signaling pathways mediated by SA act mainly during biotrophic and hemibiotrophic pathogen attack and determine the establishment of so-called systemic acquired resistance [29]. The signaling cascades mediated by JA and ET usually respond to necrotrophic pathogens, insects, herbivores and injury [30]. Extensive crosstalk between these different signal transduction pathways allows the plant to fine-tune its defenses against different types of pathogens and insect attackers.

**Figure 2:**
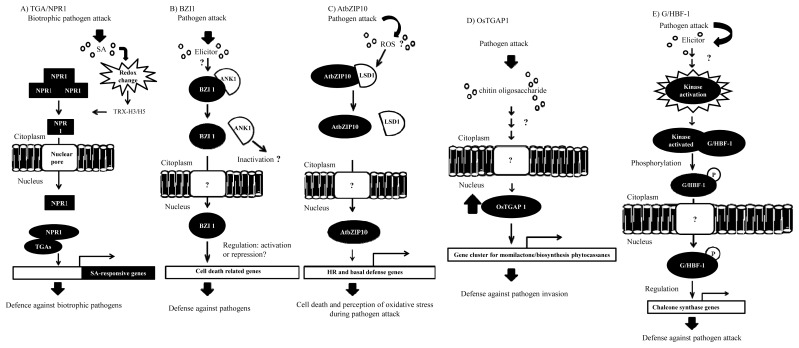
Schematic representation of the diversity of the molecular mechanisms triggered during pathogen defense responses involving bZIP proteins in different plant organisms. (**A**) The attack of a biotrophic pathogen triggers a signaling pathway mediated by salicylic acid, which alters the redox state of the cell, resulting in the dissociation of the non-expresser of PR (NPR1) protein. The monomer is translocated to the nucleus and activates the expression of SA-responsive genes by interaction with the TGACGTCA *cis*-element-binding protein (TGA) bZIP transfactors; (**B**) Recognition of elicitors after pathogen attack promotes the dissociation of the BZI1/ANK1 complex, an event that enables the entry of the BZI1 monomer into the nucleus, favoring the transcriptional regulation of cell death-related genes; (**C**) Pathogen attack activates a signaling pathway mediated by reactive oxygen species (ROS), resulting in the dissociation of the AtbZIP10/LSD1 complex. AtbZIP10 is then translocated to the nucleus to activate the transcription of HR- and basal defense-related genes; (**D**) The presence of chitin oligosaccharides induces the expression of the momilactone/phytocassane biosynthetic gene cluster through the activation of the OsTGAP1 bZIP protein; (**E**) After pathogen invasion, the recognition of elicitors induces the phosphorylation of G/HBF1, which is then translocated to the nucleus, where it regulates the expression of phenylpropanoid biosynthetic genes, such as chalcone synthase. The question marks (?) represent processes or components not yet elucidated.

Acting as regulators of SA signaling, one class of bZIP proteins that is linked to biotic stress responses comprises the TGA (TGACGTCA *cis*-element-binding proteins) [5]. In *Arabidopsis*, there are seven members of the TGA family, and these proteins play roles in plant defense, xenobiotic stress responses and development. A major advance was the discovery that TGA family members interact with an ankyrin repeat protein, non-expresser of Pathogen Related (PR) genes (NPR1), which is a key component in the SA defense signaling pathway [5] (Figure 2A). An important regulatory step in SA signaling is the nuclear translocation of NPR1 [31]. Under normal conditions, most of the NPR1 is retained in the cytoplasm as an oligomer via intermolecular disulfide bonds that are facilitated by the *S*-nitrosylation of NPR1 by *S*-nitrosoglutathione, whereby nitric oxide (NO) is covalently attached to a reactive cysteine thiol to form an S-nitrosothiol [31] (Figure 2A). Under pathogen attack, SA is synthesized and induces changes in the cellular redox state by the formation of reactive oxygen species (ROS) [32]. ROS then promote the monomerization of NPR1 through the activity of the thioredoxins H3 and H5 (TRX-H3/H5) [31] via the reduction of NPR1 by cysteine thiol-disulfide exchange [32]. In SA-induced cells, monomeric NPR1 translocates to the nucleus via nuclear pore proteins [31], and the NPR1 monomers interact with members of the TGA family (bZIP) and bind to SA-responsive gene promoters [31] (Figure 2A). During this process, NPR1 is phosphorylated and then ubiquitinated by an E3 ubiquitin ligase that has a high affinity for phosphorylated NPR1, thus targeting NPR1 for degradation by the proteasome complex [31]. This degradation of NPR1 is important for the full induction of SA-responsive target genes [31] such that NPR1 does not accumulate in the nucleus, consequently avoiding negative feedback.

With respect to crosstalk among the stress response pathways, a member of the bZIP family from pepper, CAbZIP1, is a well-documented example of bZIP participation in the integration of responses to stress. *Arabidopsis* plants overexpressing *CAbZIP1* exhibit varying degrees of growth retardation under normal conditions. Karlowski and Hirsch [14] suggested that the pleiotropic developmental phenotypes in plants may be due to defects in the ability to perceive or transmit information via hormones, as observed in previous studies [8,14]. As growth is retarded, the parallel overexpression of *CAbZIP1* is presumed to play a negative regulatory role in the development of hormone signaling, a function expected for a role in the defense against pathogens and other stress, whereby development is retarded during the adaptation to environmental stress [33].

## 3. bZIPs and *cis*-Elements Related to Pathogen Elicitors

The bZIP transcription factors related to pathogen defense recognize a diversity of *cis*-elements in the promoters of their target genes. For example, activating sequence-1 (as-1) consists of two palindromic TGACGTCA motifs separated by four nucleotides and is found in a large number of promoters induced by stress, including the PR1 (pathogenesis-related protein 1) promoter [34]. PR proteins are a diverse group of connected components of plant defense responses against pathogens [34]. Some PR proteins function as chitinases and β-1,3-glucanases with catalytic functions against fungi, whereas the functions of other PRs remain unknown [34]. Several studies show that PR proteins inhibit bacterial and fungal growth *in vitro* and that plants overexpressing these proteins have an increased tolerance to various pathogenic fungi, suggesting their role in plant defense.

The participation of the signaling molecule, salicylic acid, the bZIP transcription factor from the TGA subfamily (TGA1 a 7) [35] and the TGA-interacting protein, NPR1, is crucial for the induction of these promoters by pathogens [36,37]. Some promoters containing an as-1 element, such as glutathione-*S*-transferase F8 (GSTF8) [38], are expressed independently of NPR1 [39,40]. Another class of *cis*-elements recognized by transcriptional complexes containing bZIPs is related to the response to ET. Analyses of PR gene promoters have identified an 11 bp element responsive to ethylene TAAGAGCCGCC [9,41–43], termed the GCC box [43]. The main binding proteins for these elements are called EREBPs (ethylene-responsive element-binding proteins) and specifically bind to the GCC box [43]. The protein, EREBP, from *Arabidopsis* (AtEBP) binds specifically to a protein called *ocs*-binding factor (OBF) [44,45], which binds to a family of promoters containing a 20 bp element named *ocs*[10]. These proteins that belong to the OBF bZIP subfamily are also classified as TGA transfactors [46].

## 4. Functional Interactions of bZIPs with Other Proteins in Defense Responses

The interactions of bZIPs that modulate their activity, subcellular localization and function during the defense response against pathogens are documented in the literature [47]. It has been demonstrated that AtbZIP10 interacts *in vivo* with LSD1 (lesions simulating disease resistance 1), a protein with a zinc finger domain (Figure 2C). LSD1 is a negative regulator of cell death and protects plant cells from oxidative stress [11,48–50]. The AtbZIP10-LSD1 interaction occurs in the cytoplasm, resulting in the partial retention of AtBZIP10 [12] (Figure 2C). Furthermore, it was genetically demonstrated that AtbZIP10 is a positive regulator of the pathogen-induced hypersensitive response (HR), basal defense response and cell death induced by reactive oxygen species, activities that are antagonized by LSD1 [12] (Figure 2C).

The main protein-protein interaction documented for bZIP factors is undoubtedly the interaction between TGA factors and the NPR1 protein. NPR1 interacts with several TGA factors, including TGA2 and TGA3 [13]. TGA2 and TGA3 bind specifically to the TGACG element in the PR-1 promoter from *Arabidopsis,* and this binding is NPR1-dependent.

A yeast two-hybrid system using NPR1 as the bait revealed the isolation of four cDNAs from rice, rTGA2.1, rTGA2.2, rTGA2.3 and rLG2, which encode proteins belonging to bZIP transcription factors. The rTGA2.1, rTGA2.2 and rTGA2.3 proteins show 75%, 76% and 78% identity, respectively, with the TGA2 protein from *Arabidopsis*, the ability of which to interact with NPR1 has already been demonstrated [51]. As the basic region of the bZIP proteins are directly involved in DNA binding [52], the proteins from rice and *Arabidopsis* can recognize similar sequences of DNA. Accordingly, there are studies showing the binding of rTGA2.1 to the SA-responsive element in the *Arabidopsis* PR-1 promoter.

WRKY proteins also have members that interact with TGA proteins. In tobacco, the homologous protein, NtWRKY12, interacts both *in vitro* and *in vivo* with TGA transcription factors [15].

Studies have shown that an ankyrin-repeat protein (ANK1), an NPR1-like protein, interacts with a bZIP factor, called BZI-1 (Figure 2B). The BZI-1 protein has a DNA-binding and a D1 domain that appear to be crucial for auxin signaling and/or the defense against pathogens [8]. The molecular and functional characterization of ANK1 demonstrated that this protein is unable to bind to DNA or modulate gene transcription. Moreover, ANK1 is preferentially located in the cytosol, and its transcription is negatively regulated under pathogen attack [8]. These characteristics have led to the conclusion that ANK1 is involved in modulating auxin signaling and defense against pathogens in a manner that is dependent on its interaction with bZIP factors (Figure 2B).

## 5. bZIPs Responsive to Pathogens in Agronomic Crops

In contrast to the bZIP studies in model plants, few studies have been conducted on plants of agronomic importance. bZIPs from many other species have been studied in *Arabidopsis*, tobacco and even their corresponding orthologs, due to the difficulty of studies involving transgenic plants.

One of the first representative bZIPs studied in soybean was a G/HBF-1 protein, a bZIP member that binds to the *cis*-elements connected to pathogen elicitors, called G-box and H-box motifs. These *cis*-elements are found in the *chs15* gene promoter [16], a gene that is involved in the production of diterpenoids and flavonoids during the response to pathogens [16]. It was reported that the *G/HBF-1* transcript and protein levels were unchanged during the induction of *chs15* transcription, whereas G/HBF-1 is rapidly phosphorylated in elicited soybean cells [16] (Figure 2E).

Subsequently, a protein homologous to G/HBF-1 (SBZ1) was also characterized [53]. SBZ1 shows 63% identity in its amino acid sequence with G/HBF-1 and 60% with BZI-1 from tobacco [53]. In general, bZIP transfactors bind to *cis*-elements that have an ACGT motif [7,17], though neither G/HBF-1 nor SBZ1 bind to this sequence [16,53].

In grape, a bZIP member, named VvbZIP23, was identified as an important plant regulator of stress responses, both biotic and abiotic, and its expression was found to be strongly induced by drought, salinity, cold, ABA, ET, JA and SA [18]. A new bZIP transfactor from pepper, PPI1, which is distinct from the other bZIP members previously reported, was identified and characterized [19]. PPI1 presents a very limited homology with other bZIPs, and its expression is preferentially induced during infection, but not during the exposure to hormones related to biotic responses, such as SA, methyl jasmonate, ABA and ET [19]. This factor appears to act when infection is already established [19].

In rice, a bZIP induced by an elicitor, OsTGAP1, was found to be essential for the production of momilactones and the regulation of the expression of five genes in the momilactones biosynthesis pathway [20] (Figure 2D). OsTGAP1 was also shown to be involved in the transcriptional regulation of the genes for phytocassane biosynthesis, *OsKSL7* and *OsDXS3*[20] (Figure 2D). The overexpression of *OsTGAP1* in rice showed that this transfactor may influence the production of momilactones and phytocassanes via the upregulation of both genes under elicitor treatment. Because the expression of *OsTGAP1* is induced by treatment with chitin oligosaccharides, OsTGAP1 presents itself as a crucial regulator that controls the expression of genes involved in the biosynthesis of diterpenoids as part of the defense response of the plant and operating via the detection of chitin [20] (Figure 2D).

Two proteins in tomato, SlAREB1 and SlAREB2, have been characterized, and their expression is induced by both drought and salinity [54]. Tomato mutants overexpressing *SlAREB1* showed a greater tolerance to salt and drought stresses compared with wild-type plants [54]. Notably, many defense genes encoding proteins associated with biotic stress, such as PR proteins, protease inhibitors and catabolic enzymes, also showed increased expression during *SlAREB1* overexpression. This finding suggests that this bZIP factor is involved in the ABA signaling response to abiotic stress and possibly in response to pathogens during plant defense [54], mediating crosstalk between abiotic and biotic responses in tomato plants, similar to the bZIP protein, CAbZIP1, from pepper studied in *Arabidopsis*[33].

As many plant genomes have been completely sequenced, genome-wide identification and expression analyses of bZIP genes in various plants have been performed. Indeed, several data have been generated recently about pathogen-regulated bZIP genes using microarray, RNA-Seq, MPSS or other genome-wide expression analyses.

A cDNA microarray analysis identified 613 hot pepper genes that were transcriptionally responsive to the non-host soybean pustule pathogen, *Xanthomonas axonopodis* pv. *glycines* (Xag) [21]. Several functional types of genes were induced at the early stage of Xag infiltration [21] or downregulated at the late stage of infiltration [21]. Among these genes with altered expression, two genes encoding bZIP transcription factors showed reduced transcription in Xag-infiltrated hot peppers, suggesting that both bZIPs may serve as negative regulators in non-host defense responses of hot pepper [21].

An expression profile of more than 100 bZIP genes of maize during infection by various pathogens was obtained using DNA microarray assays [55]. Nine members of the bZIP family showed increased expression during the course of *Ustilago maydis* infection [55]. The same profile was observed during infection by *Colletotrichum graminicola*, and it was observed that the expression of the ZmbZIP65, 21 and 53 genes was increased after 96 h of infection [55]. Two cocoa bZIPs (RT42C09 and RT57A09) were studied according to their expression during *Moniliophthora perniciosa* infection, with expression increasing after the establishment of infection [56].

Other genome-wide studies with no functional characterization of bZIP genes have been conducted in different organisms [57,58]. A microarray experiment performed to reveal the molecular mechanisms underlying the hypersensitive reaction (HR) of rice to *Xanthomonas oryzae* pv. *oryzicola* (Xoc) mediated by a maize NBS-LRR type R gene, *Rxo1*, resulted in the differential expression of 83 transcription factor (TF) genes in most TF families. bZIP transcription factors were included as some of the TF genes showing altered expression in this study [57]. The incompatible pathosystem between resistant cotton (*Gossypium barbadense* cv. 7124) and *Verticillium dahlia* strain V991 was used to study the alterations in the cotton transcriptome after pathogen inoculation using RNA-Seq [58]. Of the 32,774 genes detected by mapping the tags to the assembly cotton contigs, 3442 defense-responsive genes were identified, with several TFs among them, including bZIP genes [58]. The majority of TFs were suppressed based on the RNA-Seq analysis after pathogen inoculation, which moderately correlated with the results obtained by qPCR [58].

## 6. Conclusions

Understanding the molecular mechanisms of plant responses to biotic stress caused by pathogens is very important, as it facilitates the exploitation of these mechanisms to improve stress tolerance and productivity. This review summarizes the role of important plant bZIP transcription factors in pathogen stress responses. Due to the use of new technologies and diverse approaches, including physiology, biochemistry, molecular biology and computational biology, the knowledge of plant signal transduction pathways has rapidly progressed, contributing to the understanding of the various aspects of plant biotic stress responses. Much progress in bZIP transcription factor research has been made over the past 20 years. However, most of the advances are related to the involvement of these proteins in abiotic stress and in seed and plant development, and there are few examples of functional research on biotic stress, particularly with regard to major cultivars. Furthermore, considering the size of this gene family, the identification of the bZIP function in biotic stress will remain a major challenge in the coming years. To achieve a better understanding of their role during biotic stress, it is of vital importance to identify the interacting partner of the bZIP proteins that cooperate to regulate the transcription of downstream target genes under certain conditions. It is also important to determine the key components of the signal transduction pathways through which they physically interact. A functional analysis of these bZIPs will thus provide more information on the intricate regulatory networks involved in biotic stress responses and the crosstalk between different signaling pathways during stress adaptation. Furthermore, considering bZIPs as candidate genes in breeding and other crop improvement programs will provide a clear understanding of biotic stress-related signal transduction events and will eventually lead to the development of genetically manipulated crop varieties with improved stress tolerance.

## Figures and Tables

**Table 1:** Function of basic leucine zipper (bZIP) transcription factors in biotic stress. HR, hypersensitive response; SA, salicylic acid.

Protein	Organism	*cis*-Element	Inducers	Function in plant defense	References
CabZIP1	*Capsicum annuum* L.	G-box	ET, SA, MeJA and pathogen infection	Decrease in plant growth rates	[8]
OBF PROTEIN	*Arabidopsis thaliana*	*osc* element	SA	Induction of PR gene expression	[9,10]
AtbZIP10	*Arabidopsis thaliana*	G-box and C-box	Uncharacterized	Positive regulator of HR, cell death and basal defense response	[11]
TGA MEMBERS	*Arabidopsis thaliana*	TGACG element	SA	Induction of PR gene expression	[12]

rTGA2.1, rTGA2.2, rTGA2.3	*Oryza sativa*	PR-1 promoter (in *Arabidopsis*)	SA	Induction of SA responsive gene expression	[13]

BZI-1	*Nicotiana tabacum*	ACEs	Uncharacterized	Auxin signaling and plant defense	[14]
G/HBF-1	*Glycine max*	G-box and H-box	Uncharacterized	Regulation of defense gene expression	[15]
SBZ1	*Glycine max*	G-box and H-box	Uncharacterized	Regulation of defense gene expression	[16]
VvbZIP23	*Vitis vinifera*	Uncharacterized	ABA, ET, JA and SA.	Regulation of biotic and abiotic stress responses	[17]
PPI1	*Capsicum chinense*	ACEs	Pathogen infection	Regulation of defense gene expression	[18]
OsTGAP1	*Oryza sativa*	TGACG element	Chitin oligosaccharides	Regulation of genes involved in the biosynthesis of diterpenoids	[19]
SlAREB1	*Solanum lycopersicum*	ABRE (ABA-responsive element)	ABA	Pathogens response	[20]
RT42C09	*Theobroma cacao*	Uncharacterized	Pathogen infection	Pathogens response	[21]
RT57A09	*Theobroma cacao*	Uncharacterized	Pathogen infection	Pathogens response	[21]
